# Recent advances in understanding the extracellular calcium-sensing receptor

**DOI:** 10.12688/f1000research.8963.1

**Published:** 2016-10-19

**Authors:** Matilde Colella, Andrea Gerbino, Aldebaran M. Hofer, Silvana Curci

**Affiliations:** 1 Department of Biosciences, Biotechnology and Biopharmaceutics, University of Bari , Bari, Italy; 2 Department of Surgery, Brigham & Women’s Hospital, Harvard Medical School and VA Boston Healthcare System, West Roxbury, MA, USA

**Keywords:** extracellular Ca2+ sensor, CaR, receptor trafficking

## Abstract

The extracellular calcium-sensing receptor (CaR), a ubiquitous class C G-protein-coupled receptor (GPCR), is responsible for the control of calcium homeostasis in body fluids. It integrates information about external Ca ^2+^ and a surfeit of other endogenous ligands into multiple intracellular signals, but how is this achieved? This review will focus on some of the exciting concepts in CaR signaling and pharmacology that have emerged in the last few years.

## Introduction

Alteration in the activity or function of the extracellular Ca ^2+^ (Ca ^2+^_ext_)-sensing receptor (CaR; also named CaSR or CaS) is linked to several genetic disorders of calcium homeostasis ^1^, such as familial hypocalciuric hypercalcemia (FHH) and neonatal severe hyperparathyroidism (NSHPT) ^2^, both caused by loss-of-function mutations of the CaR gene, and those that occur as a consequence of gain-of function mutations of the CaR, e.g., autosomal dominant hypocalcemia (ADH) and Bartter syndrome (BS) type V ^3– 5^. However, the CaR is also a factor in other more common pathologies that include chronic kidney disease ^6^, cancer ^7^, cardiovascular pathologies ^8– 11^, and Alzheimer’s disease ^12^. For a complete survey of CaR’s function in molecular physiology and pathology, readers are referred to some of the many recent reviews on the topic ^13^.

**Figure 1.**
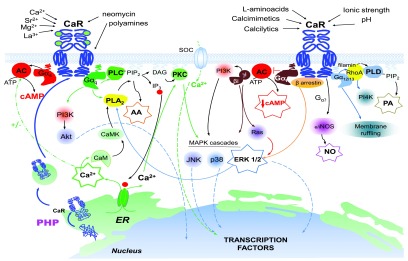
Signal transduction mediated by the extracellular calcium-sensing receptor (CaR). Schematic of the dimeric extracellular CaR at the plasma membrane. A complex network of intracellular transduction cascades is activated by numerous orthosteric agonists or allosteric modulators converging either on the bi-lobed venus-flytrap domain or on the seven transmembrane domain of the CaR. For clarity, two G-protein-coupled receptors (GPCRs) are shown; this is not meant to imply that the ligands depicted are linked preferentially to a particular intracellular signaling pathway, although see section in text on biased agonism. Abbreviations: AA, arachidonic acid; AC, adenylate cyclase; Akt, protein kinase B; ATP, adenosine triphosphate; CaM, calmodulin; CaMK, Ca ^2+^/calmodulin-dependent protein kinase; cAMP, cyclic AMP; DAG, diacylglycerol; eNOS, endothelial nitric oxide synthase; ER, endoplasmic reticulum; ERK 1/2, extracellullar-signal-regulated kinase; Gα _s_, Gα _i_, Gα _q_, Gα _12/13_, α subunits of the s-, i-, q-, and 12/13-type heterotrimeric G-proteins, respectively; iNOS, inducible nitric oxide synthase; IP _3_, inositol-1,4,5-trisphosphate; JNK, Jun amino-terminal kinase; MAPK, mitogen-activated protein kinase; MEK, MAPK kinase; NO, nitric oxide; p38, p38 mitogen-activated protein kinase; PA, phosphatidic acid; PHP, pharmacoperones; PI3K, phosphatidylinositol 3-kinase; PI4K, phosphatidylinositol 4-kinase; PIP _2_, phosphatidylinositol 4,5-bisphosphate; PKC, protein kinase C; PLA _2_, phospholipase A2; PLC, phospholipase C; PLD, phospholipase D; RhoA, Ras homolog gene family, member A; SOC, store-operated Ca ^2+^ channel.

We will first address when and how Ca ^2+^_ext_, the primary ligand for the CaR, changes in tissue spaces. Ca ^2+^ is, however, just one of the many activators of this fascinating receptor; the CaR is “built” to interact with a dizzying array of other orthosteric agonists and also allosteric modulators that influence the receptor’s response to calcium ions ( Table 1). These endogenous ligands activate multiple intracellular signaling pathways, often in the same cell type ( Figure 1). However, the CaR can discriminate between its ligands to preferentially activate a particular subset of signaling pathways at the exclusion of others through the phenomenon known as biased agonism. In addition, CaR signaling can be dynamically regulated through agonist-dependent trafficking of intracellular receptors to alter the net amount of the receptor at the plasma membrane. We will address how certain ligands act as “pharmacoperones” to shepherd the receptor to the cell surface. All of these factors serve to fine-tune the activity of the receptor. Finally, we discuss the incredible potential of this newfound information to aid in the design of novel, smarter, drugs able to rescue mutated receptor mislocalization and function, and bias CaR-mediated signaling towards particular pathways.

**Table 1.** Principal orthosteric agonists and allosteric modulators of the calcium-sensing receptor.

Orthosteric agonists (type I calcimimetics)		References
Inorganic divalent and trivalent cations	***High potency:*** Gd ^3+^; Eu ^3+^; Tb ^3+^ ***Intermediate potency:*** Zn ^2+^; Ni ^2+^; Cd ^2+^; Pb ^2+^; Co ^2+^; Fe ^2+^ ***Low potency:*** Ca ^2+^; Mg ^2+^; Ba ^2+^; Sr ^2+^; Mn ^2+^	125– 128
Polyamines	Spermine, spermidine, putrescine	129
Aminoglycoside antibiotics	Neomycin, gentamycin, tobramycin, poromomycin, kanamycin, ribostamycin	130– 132
Basic polypeptides	Poly-l-arginine, poly-l-lysine, protamine, amyloid β-peptides	133– 135
**Allosteric modulators** **(type II calcimimetics)**		
L-amino acids	Phenylalanine, tryptophan, tyrosine, histidine	136– 138
Glutathione analogs	γ-glutamyl-tripeptides: glutathione, S-methylglutathione, S-propylglutathione γ-glutamyl-tripeptides: γ-Glu-Ala, γ-Glu-Cys	139– 140
Small molecule calcimimetics	***First generation:*** NPS R-568, NPS R-467	141, 142
	***Second generation:*** cinacalcet	143– 145
	***Third generation:*** dibenzylamine calcimimetics, R,R-calcimimetic B, AC-265347	94, 146, 147
Small molecule calcilytics	NPS 2143, Calhex 231, ATF936, AXT914, ronacaleret, NPSP795, SB-423557, SB-423562	97, 142, 148– 150

## Extracellular Ca ^2+^ fluctuations and Ca ^2+^ microdomains

Systemic Ca ^2+^ levels (~1.1–1.3 mM) are under stringent homeostatic control exerted by organs such as the parathyroid glands, bone, renal system, and intestine ^14^. Nonetheless, local fluctuations in Ca ^2+^_ext_ levels have been identified and characterized in the restricted volume of interstitial fluids bathing cells of many tissues ^15^. The amplitude and shape of these Ca ^2+^_ext_ fluctuations is thought to represent an autocrine/paracrine form of cell-to-cell communication. Pharmacological agents directed at the CaR therefore work upon a complex backdrop of changing external [Ca ^2+^]. This has the potential to markedly affect the way in which a drug (particularly those in the class of the allosteric modulators) acts on the receptor in any given moment. Knowledge about these local fluctuations in calcium remains, arguably, among the most significant barriers to fully understanding CaR pharmacology *in vivo*.

Many factors are believed to participate in the generation of physiologically relevant Ca ^2+^_ext_ changes, e.g. a) intracellular Ca ^2+^ signaling events, b) Ca ^2+^ extrusion via discharge of calcium-enriched granules, and c) synchronous opening of voltage-operated Ca ^2+^ channels. Below, we describe briefly how these extracellular microdomains can be measured and give examples of how they are generated.

### Measuring extracellular Ca ^2+^ levels

Historically, real-time measurements of Ca ^2+^_ext_ changes in close proximity to the plasma membrane have been hampered by the lack of proper experimental tools to physically access these restricted compartments in intact tissues and by difficulties in measuring [Ca ^2+^] fluctuations against the background of mM Ca ^2+^ concentrations normally present outside the cell. Although many different experimental approaches have been proposed to quantify Ca ^2+^_ext_ fluctuations in a number of diverse tissue models, each of them presents limitations with regard to either sensitivity or spatial resolution. For example, Ca ^2+^-sensitive small molecule fluorescent indicators have proven useful to visualize the temporal/spatial dynamics of Ca ^2+^_ext_ changes, since they provide sensitivity, time resolution, and access to limited spaces ^16– 24^. However, these methods require experiments to be performed in non-physiological conditions such as low or nominally free Ca ^2+^_ext_ because of the relatively high Ca ^2+^ affinity of the available fluorophores. For example, Tepikin and Petersen introduced the droplet technique ^25– 27^ to reliably quantify Ca ^2+^_ext_ changes induced by active Ca ^2+^ extrusion through the plasma membrane Ca ^2+^-ATPase (PMCA) of acinar cells. Fluo-3 was used to characterize changes in Ca ^2+^_ext_ in small clusters of exocrine gland cells maintained in a tiny droplet of solution covered with oil to prevent evaporation, but this method could only be used under Ca ^2+^-free media conditions on account of the high affinity of the Ca ^2+^ indicator.

We, as well as others, have used Ca ^2+^-selective microelectrodes extensively to directly record the profile of changes in Ca ^2+^_ext_ in the restricted domains of different experimental tissue models following Ca ^2+^-mobilizing stimulation ^28– 38^. As described further below, we also used Ca ^2+^-sensitive microelectrodes to measure real-time Ca ^2+^_ext_ changes induced by the glucose-dependent discharge of Ca ^2+^-rich insulin granules ^39^. Ion-sensitive microelectrodes present certain advantages. First, measurements of Ca ^2+^_ex_ changes under physiological conditions are allowed owing to the availability of Ca ^2+^-sensitive resins with affinities in the μM and mM range. In addition, it is possible to record Ca ^2+^_ext_ changes for hours without technical drawbacks such as the bleaching of fluorescent indicators. However, this approach requires a high level of patience and expertise and samples only one small region of the tissue, so it is not amenable to high-throughput measurements. Moreover, it is difficult to execute in many tissue types. This is an arena in which further developments would be welcome.

### Origins of extracellular Ca ^2+^ microdomains

***Intracellular Ca ^2+^ signaling events.*** Cells facing restricted diffusion spaces can experience Ca ^2+^_ext_ fluctuations during intracellular Ca ^2+^ signaling events as a result of activation of Ca ^2+^ efflux (e.g. by PMCA and/or Na ^+^/Ca ^2+^ exchanger) and influx (e.g. by store-operated channels [SOCs]) across the plasma membrane. The genesis of significant Ca ^2+^_ext_ microdomains requires either differential dynamics or polarized asymmetry of Ca ^2+^ influx/efflux mechanisms ^40– 43^. For example, we found that stimulation with Ca ^2+^-mobilizing agonists resulted in substantial local increase in Ca ^2+^_ext_ at the luminal face and a comparable depletion at the serosal aspect of gastric acid-secreting cells ^38^. An increase in [Ca ^2+^] in the gastric gland lumen is due to activation of Ca ^2+^-ATPase, which is highly expressed at the apical membrane of these cells, where it co-localizes with CaR ^38^.

***Ca ^2+^ extrusion via discharge of calcium-enriched granules.*** Very high Ca ^2+^ concentrations have been measured within secretory granules ^44– 46^. For example, insulin granules from rat insulinoma have a granular concentration of Ca ^2+^ between 60 and 120 mM ^47^. Therefore, one can assume that exocytotic events may generate consistent increases in Ca ^2+^_ext_. Recently, we showed that the stimulation of insulin secretion by high glucose and other secretagogues resulted in late elevation of Ca ^2+^_ext_ within rat insulinoma (INS-1E) β-cell pseudoislets, as measured with Ca ^2+^ microelectrodes ^39^. Ca ^2+^ extrusion via Ca ^2+^-enriched granules has also been proposed for a number of different cell types that undergo exocytosis such as salivary gland cells ^22^, bovine adrenal medullary cells ^48^, neurohypophyseal nerve endings ^49^, and sea urchin eggs ^50^.

***Synchronous opening of voltage-operated Ca ^2+^ channels.*** Excitable cells have, in addition to the above-mentioned mechanisms, a variety of voltage-dependent Ca ^2+^ entry pathways that might impact Ca ^2+^_ext_ during their physiological activity ^51– 53^. In the central nervous system, synchronous opening of voltage-gated Ca ^2+^ channels (VGCCs) can stimulate significant reductions in Ca ^2+^_ext_^54, 55^. Pumain and Heinemann recorded Ca ^2+^_ext_ reductions from a basal level of 1.25 mM to as low as 0.08 mM in rat neocortex following the application of excitatory amino acids ^54^. In cardiac muscle, transient depletions in Ca ^2+^_ext_ by about 200 μM were measured during a single heartbeat ^16^. In mouse islets of Langerhans and INS-1E pseudoislets, glucose stimulation induced a reversible and significant depletion in Ca ^2+^_ext_ by about 500 μM as a consequence of VGCC-mediated Ca ^2+^ influx across the plasma membrane ^30, 33, 39^.

## New paradigms in extracellular calcium-sensing receptor trafficking and signaling pave the way for the design of novel, smart drugs

In the classical (yet oversimplified) view, upon interaction with a G-protein-coupled receptor (GPCR), ligands stabilize preferred conformational state(s) that in turn activate distinct subsets of G-protein-mediated downstream signaling pathways ^56– 58^. When GPCRs are coupled to multiple G-proteins in the same cell type, as is the CaR, the old dogma hypothesized that they activate each of the downstream signals equally, without preference for any one pathway ^58– 61^.

In the past few years, several studies have painted a more complex scenario, in which receptors, existing in multiple active states, can specifically trigger selected pathways at the exclusion of others ^62^. This will depend not only on the signaling toolkit of the cell in which they are expressed but also on numerous other factors ^63^, such as the localization of the GPCRs, the duration of stimulus for GPCRs working in non-equilibrium conditions, the downstream signaling protein level (i.e. involvement of different effectors able to shape diverse Ca ^2+^ and cAMP microdomains and kinetics), and the specific agonist/modulator activating the receptor ^64^. Also, it has been shown that GPCRs traffic through subcellular compartments such as the nucleus ^65^, mitochondria ^66^, and endosomes ^67, 68^, where they are capable of initiating specific signaling pathways.

In this context, pharmacological studies have shown that ligands are able to bias the signaling of their GPCRs towards specific intracellular responses and/or are capable of crossing cell membranes, thus activating or rescuing intracellular GPCRs (by acting as molecular chaperones) ^69^. The development of new technologies, such as microscopy techniques and probes to follow receptor trafficking ^63, 70^ and to assess in real time subcellular signaling dynamics ^71, 72^ as well as biased signaling ^73^, has been essential for such advances and will certainly continue to promote novel and exciting discoveries in this field.

## The “anti-conformist” extracellular calcium-sensing receptor traffics to the plasma membrane via a novel route: agonist-driven insertional signaling

In the classical life cycle of GPCRs, the newly synthesized receptor is inserted into the endoplasmic reticulum and, after folding, is transported through the cis-Golgi/Golgi/trans-Golgi, where it goes through further post-translational changes. Then the mature protein, packaged in small vesicles, undergoes insertion into the cell membrane. If misfolded, the protein is degraded by the proteasome. Upon binding, ligands stabilize preferred conformational state(s) of the receptor that initiate intracellular signaling. The process is terminated via receptor internalization mediated by GPCR kinase (GRK) phosphorylation and β-arrestin(s) recruitment ^74^. The internalized receptor can be degraded by the lysosome or recycled to the cell membrane. Importantly, both β-arrestin and internalized receptors can initiate signaling.

It is well established that the fine balance among maturation, internalization, recycling, and degradation can influence the net amount of cell surface receptor level and thus represents a mechanism for the cell to regulate receptor sensitization and modulate the strength of signal transduction ^75^. The intensity of signaling is thus related to the quantity of GPCRs expressed on the cell surface and accessible for ligand stimulation. This is also true for the CaR, as recently demonstrated by Brennan and colleagues ^76^.

Relevant advancements in the knowledge of the key players involved in CaR biosynthesis and trafficking have been achieved in the last ten years ^77– 81^. Both early and recent studies have highlighted that two hallmarks of the CaR are the negligible functional desensitization and the existence of a significant amount of CaR in intracellular membranes. Early studies indicated, both by western blotting or immunohistochemistry ^14, 82, 83^, that CaR immunoreactivity reflected a predominantly intracellular, core-glycosylated form. It is now becoming clear that such an observation is not a mere artifact but is strictly related, and even of functional importance, to the complex and mutual interaction between CaR trafficking and signaling. In fact, both minimal desensitization and high levels of intracellular CaR can be explained by the model of agonist-driven insertional signaling (ADIS) ^78, 84^.

The process of ADIS depends upon the regulated release of mature CaR proteins from a large intracellular pool located in the endoplasmic reticulum and Golgi/post-Golgi vesicles. The rate of CaR plasma membrane insertion increases as a function of the concentration of CaR agonists and/or allosteric modulators, while the receptor already at the plasma membrane undergoes constitutive endocytosis without substantial recycling. Importantly, and predictably, in this model, CaR signaling can be dynamically regulated by the trafficking of intracellular CaR to the plasma membrane through an agonist-dependent modulation of the net amount of CaR at the plasma membrane. This has implications in both health and disease ^85^.

## New insights into the mechanisms underlying the therapeutic potential of allosteric modulators of the extracellular calcium-sensing receptor

As summarized in Table 1, besides the orthosteric ligands, which upon binding to agonist-binding sites are able to stimulate the receptor in the absence of Ca ^2+^ (or any other ligand), the other class of CaR agonists is represented by allosteric modulators, which after binding to different sites alter the receptor conformation and, as a consequence, affect receptor responses to orthosteric ligands. This action can be exerted in a positive (calcimimetics) or a negative (calcilytics [ Table 1]) direction.

Interestingly, a number of recent reports have shown that allosteric modulators can act as pharmacoperones. Pharmacoperones (or pharmacological chaperones or pharmacochaperones) are membrane-permeant ligands (agonists, antagonists, or allosteric modulators) that reach the misfolded protein at the site of its biosynthesis and trafficking (most frequently the endoplasmic reticulum) and, by stabilizing the receptor structure, rescue the protein to the cell surface ^69^.

Breitwieser's group has published a number of interesting papers highlighting the capability of CaR allosteric modulators to function as pharmacoperones ^86– 88^. While an early study reported the synergistic effect of acute treatment with L-phenylalanine and NPS R-467 on CaRs with inactivating mutations ^89^, Breitwieser and colleagues first showed that overnight treatment of HEK293 cells expressing loss-of-function mutant CaRs with the calcimimetic NPS R-568 rescued plasma membrane expression and signaling in 50% of the mutations examined ^86, 87^. Similar results were obtained by other groups, although the authors did not investigate the cell surface expression of CaR after NPS R-568-mediated signal rescue ^90, 91^. Interestingly, the capability of the calcimimetic NPS R-568 to rescue CaR activation without altering the cell surface expression of the mutant proteins was shown in a recent study ^92^, suggesting a mutant-specific effect of this drug as a pharmacoperone.

Relevant findings in this area have also been provided by Leach and colleagues ^93, 94^. They showed that calcimimetics, including the only calcimimetic approved in the clinic (cinacalcet), effectively rescue trafficking and signaling of CaR mutants exhibiting a loss of cell surface expression. They also found that the calcilytic NPS 2143 effectively promotes trafficking of CaR mutants to the cell membrane while negatively modulating CaR signaling ^93, 95^. This is in contrast to other studies with NPS 2143 showing a reduced ^86^ or unchanged ^92^ effect on the expression of diverse CaR gain-of-function mutants, suggesting that a mutant-specific pharmacoperone effect also exists for NPS 2143.

The potential of calcilytics for patients with activating CaR mutations has been further examined *in vitro* ^96^. More recently, the new quinazolinone-derived calcilytics were shown to be effective in attenuating enhanced calcium signaling in mutations causing BS and ADH ^97^. NPS 2143 was also found to correct signaling defects in HEK293 cells transfected with Gα11-mutated proteins causing ADH2 and uveal melanoma ^98^. Very interestingly, the effectiveness of both old (i.e. NPS 2143) ^99^ and new (i.e. JTT-305/MK-5442) ^100^ calcilytics was recently assessed *in vivo* in mouse models harboring ADH gain-of-function CaR mutations.

## Biased signaling at the extracellular calcium-sensing receptor

Recent reports suggest that the therapeutic potential of new classes of CaR modulators, as well as the pathophysiological role of endogenous agonists, could be further improved by exploiting the phenomenon of biased signaling ^11^. Biased signaling (also known as ligand-directed signaling, stimulus bias, biased agonism, or functional selectivity) ^62, 101, 102^ represents a general, albeit only recently appreciated, signaling characteristic of GPCRs ^58^. It refers to the ability of different ligands to stabilize distinct receptor conformations and preferentially direct GPCR signaling towards a specific set of pathways while excluding/reducing others.

This concept, while greatly complicating the scenario of GPCR signaling, opens up new perspectives in the design of smart and tissue-specific drugs ^103^. The existence of ligand- and tissue-specific effects in the signaling pathways activated by the CaR, although not precisely quantified, is traceable in a vast number of papers published throughout the years. In fact, in many cases, biased signaling at the CaR might have been underestimated owing to the use of single assays for the evaluation of CaR signaling outputs (most commonly cytosolic calcium dynamics) or the low number of CaR agonists and modulators tested.

A peculiar case of biased signaling at the CaR was observed in response to an allosteric autoantibody isolated from a patient with acquired hypocalciuric hypocalcemia. The antibody potentiated the effects of Ca ^2+^_ext_ via Gq signaling while suppressing Gi-mediated signaling ^104^. In other examples, Bruce and colleagues reported differential effects of CaR agonists on Ca ^2+^ dynamics in isolated acini and interlobular ducts of rat pancreas ^105^. Ziegelstein showed that in human aortic endothelial cells only spermine was able to induce intracellular Ca ^2+^ release and nitric oxide production, whereas Ca ^2+^_ext_, Gd ^3+^, and neomycin were ineffective ^106^. Furthermore, Smajilovic *et al*. demonstrated a concentration-dependent vasodilatation in rat aorta with the addition of cinacalcet, whereas the agonists neomycin and Gd ^3+^ were ineffective ^107^.

On these bases, in the last three years, an increasing number of reports have focused on the physiological and pathological role of biased signaling exerted on the CaR by its physiological agonists and pharmacological modulators as well as on mutation-dependent alterations in such bias. A key contribution to this field comes from the group of Bräuner-Osborne ^108– 110^. By exploring the effect of 12 orthosteric CaR agonists on inositol (1,4,5)-trisphosphate (IP _3_) accumulation, cAMP inhibition, and ERK1/2 phosphorylation in HEK293 cells stably transfected with rat CaR, Thomsen and colleagues ^110^ revealed that Ca ^2+^_ext_ is biased towards cAMP inhibition and IP _3_ accumulation, while spermine shows a strong bias towards ERK1/2 phosphorylation. Also, this study demonstrated for the first time that ERK1/2 is partially activated through the recruitment of β-arrestin by the CaR. The same group also obtained interesting results concerning strontium ranelate, currently used in the clinic for the treatment of osteoporosis ^109^. As previously suggested by Chattopadhyay and colleagues ^111^, and contrary to the results obtained by Coulombe ^112^, Sr ^2+^ was shown to bias CaR signaling towards ERK1/2 in rat medullary thyroid carcinoma 6–23 cells. Also, in rabbit osteoclasts, while both Sr ^2+^ and Ca ^2+^ produced stimulation of PLC and translocation of NF-kB, in contrast to Ca ^2+^_ext_, Sr ^2+^ signaling was independent of the IP _3_ pathway and induced apoptosis via PKC activation ^113^.

The possibility of exploiting biased agonism at the CaR has been extensively explored by the group of Christopoulos and Leach ^93, 114– 116^. These authors analyzed the effect of calcimimetics and calcilytics on a number of CaR mutations ^115^ (reviewed in 95, 103), demonstrating that mutated CaR proteins can display altered signaling bias. Importantly, and as stated above, both cinacalcet and NPS 2143 were shown to effectively rescue mutants to the cell membrane, with a bias of both compounds toward the modulation of agonist-stimulated Ca ^2+^ mobilization ^93^. There is no doubt that these results have relevant therapeutic potential.

To date, cinacalcet has been used for the treatment of hyperparathyroidism and to correct Ca ^2+^_ext_ in patients with loss-of-function CaR mutations. However, because of its hypocalcemic side effects, presumably due to CaR-mediated calcium-dependent calcitonin secretion from thyroid parafollicular C-cells ^108^ and potentiation of renal CaRs, its use has been restricted to patients with end-stage renal disease. Thus, a drug that suppresses PTH secretion without raising serum calcitonin would be therapeutically advantageous.

Potential clues towards the search for a calcimimetic with low/no effect on calcitonin was hinted at in a very recent paper ^94^. In this work, the authors demonstrated that while phenylalkylamine calcimimetics were biased towards Ca ^2+^ mobilization and IP _1_ accumulation (a stable metabolite of IP _3_), R,R-calcimimetic B and AC-265347 biased CaR signaling towards pERK1/2 and IP _1_ accumulation. This finding may explain the preference of R,R-calcimimetic B and AC-265347 for the suppression of PTH release versus the stimulation of calcitonin secretion *in vivo.*

## Structure-function relationships and future prospects

Recent work explored the structural requirements for bias and allostery mediated by old and new classes of positive and negative allosteric modulators of the CaR ^116^. Further, Jenny Yang’s lab has published several papers about the potential Ca ^2+^ binding sites and their relevance for related diseases ^117– 124^. Recently, these authors solved the first high-resolution crystal structure of the ECD of human CaR bound with Mg ^2+^ ^117^. Of note, a high-affinity tryptophan derivative was found in the crystal structure of the CaR that seems to play a role in potentiating the function of the receptor ^117^. These studies represent important progress in the field, since they provide new insights into the structural basis of human diseases arising from CaR mutations. Ultimately, the subtle differences in modulator binding sites revealed by structural studies may be exploited to design drugs able to elicit distinct signaling outcomes and thus be effective on specific mutations (patient-specific drugs) and/or on tissue-specific signaling pathways (tissue-specific drugs).

In this scenario, a fundamental challenge for future research will be to set up methodological tools to validate these latest pharmacological advances in more physiologically relevant models, such as primary cells or animal models. It also remains to be seen how the functional effects of these drugs are altered in the complex landscape of changing [Ca ^2+^] in extracellular microdomains *in vivo*.
